# Medical obituaries and compulsive reading

**DOI:** 10.1192/bjb.2025.10194

**Published:** 2026-08

**Authors:** Claire Hilton

**Affiliations:** Royal College of Psychiatrists, London, UK

**Keywords:** History of psychiatry, qualitative research, ethics, education and training, quality of life

## Abstract

Medical Royal Colleges publish obituaries to record and celebrate the lives of colleagues after their deaths. Who is included in this roll of honour, the preferred literary style, and the organisation of the commissioning and publishing all vary between colleges. Since obituaries have fashions, shaped by culture and practical considerations, it is worthwhile, from time to time, for institutions to review the approaches they take. This paper draws on practices past and present, including those of national newspapers and Royal Colleges, to stimulate further discussion on the subject.

After leading old age psychiatrist Dr Felix Post died in 2001 there was uproar among his colleagues because this journal did not publish an obituary. Subsequently, six of them wrote separate brief tributes, which appeared in the journal under the title ‘Remembering Felix Post’.^1^ Getting obituaries wrong, whether in timeliness, content or choice of who to include, may be distressing to those whose lives have been influenced by the deceased.

Many of us will have tried to grasp what makes someone tick, the various influences on them, the decisions they make and the things they do. This study considers that from the point of view of creating obituaries to record and celebrate the lives of colleagues after their deaths. It was carried out after I was appointed obituaries editor for the *BJPsych Bulletin*. It aims to open discussion about the ways in which the Royal College of Psychiatrists (RCPsych) records and remembers the lives of former colleagues across all membership categories.^2^

The first ‘obituary’ in the *Asylum Journal*, the predecessor of the *British Journal of Psychiatry* (*BJPsych*) and *BJPsych Bulletin*, in February 1854 read as follows: ‘On the 12th instant, at College Gardens, Gloucester, the Rev. Thomas Evans, D.D., for upwards of twenty years Chaplain to the Gloucestershire General Lunatic Asylum’.^3^ The second, in October 1854, read: ‘J.H.B. Sandon, Esq., late Medical Superintendent of the Dorsetshire County Asylum, at the latter end of August, of phthisis’.^4^ As well as marking their passing and memorialising them in a professional context, both raise questions about psychiatry at the time. Why, for example, was a hospital chaplain included, and did Dr Sandon (recently appointed to his post, about 30 years old and buried in Charminster alongside his patients^5^) catch phthisis (pulmonary tuberculosis) in the course of his clinical work?

The term ‘obituary’ is used in many ways, from death notifications – similar to the *Asylum Journal* entries – to full page articles in broadsheet newspapers, but rarely more. The briefest, usually soon after someone has died, inform readers that the death has occurred, and may be a call for people to attend a funeral, offer condolences and support the bereaved. Some medical Royal Colleges, including the RCPsych, have an online *in memoriam* list.^6,7^ Others have (re)-introduced a list into a journal or magazine, such as the *Bulletin of the Royal College of Surgeons of England* (every two months) and *BJPsych Bulletin* (annually; under review). Arguably, time is of the essence for these lists if they are to permit or encourage personal contact with the bereaved in a timely manner.

The sort of obituaries this paper aims to consider are longer tributes about the lives of the deceased and their contributions to society – hopefully positive but not necessarily – in their professional capacity and beyond. Typically, each is 400 to 1000 words, written shortly after the death, but nevertheless well researched. For the most eminent in society, to ensure prompt publication, the press commissions obituaries while the subject is still alive, and sometimes the author predeceases the person being commemorated.^8^ Currently, the *BJPsych Bulletin* has a small page allocation for these tributes, for people who fulfil the criterion of having ‘made a significant contribution to the specialty, or related fields, at national or international level’.^9^ Separate from the *BJPsych Bulletin* and organised by the RCPsych is a small online obituaries archive. Wendy Burn initiated this in 2019 while RCPsych President, advocating that more psychiatrists should be recognised and remembered for their life’s work. However, as of June 2025, only about one in ten of deaths of members notified to the RCPsych had been commemorated there.^7^

Psychiatrists should be particularly good at writing obituaries. A fascinating and integral part of our careers is working holistically with our patients, a biopsychosocial model that takes their life story into account.^10^ We also encounter people suffering prolonged, complicated grief reactions, and may have our own personal experiences of loss. All these should help us create obituaries that are meaningful, if not therapeutic, to bereaved relatives, to colleagues and to others. For the relatives, public acknowledgment of the life, death and legacy of a recently deceased individual may help ease grief. Funeral orations and memorial services are part of that, but they are ephemeral, whereas obituaries are intended to endure. Obituaries may contribute to public perceptions of a medical specialty, inspire students to enter the profession (‘I’d like to do that job’), promote role models (‘I’d like to conduct my life like that person’) and contribute to the heritage and historical understanding of a profession.

Since obituaries have fashions, shaped by culture, values and practicalities,^11^ it is legitimate for medical Royal Colleges to examine their approaches to them, from time to time. To assist the RCPsych, should it wish to do that, it seemed appropriate to try to understand what other medical Royal Colleges do. Sonia Walter, the RCPsych’s Chief Executive Officer, sent a brief questionnaire to her Royal College counterparts asking how they manage the practicalities and processes of creating obituaries of their members. Themes included: how each college decides whose obituaries to publish; how the obituaries are obtained; whether they are published on the website, or in a journal/magazine, or both; if both, then how the editorial process is coordinated. Of the 13 Royal Colleges contacted, 7 responded. Others give ample information on their websites. The 10 that provided information used in this study are listed in Box 1 (with their abbreviations), but to avoid being judgemental, some questionnaire responses are not directly attributed in the text.

Box 1 Names (and abbreviations) of 10 medical Royal Colleges providing information used in this study about their obituary practices

Information derived from website and response to questionnaire:

- Royal Australian and New Zealand College of Psychiatrists (RANZCP)
- Royal College of Emergency Medicine (RCEM)
- Royal College of Physicians of London (RCP)
- Royal College of Physicians of Edinburgh (RCPE)
- Royal College of Radiologists (RCR)
- Royal College of Surgeons of England (RCS)
- Royal College of Surgeons of Edinburgh (RCSEd)

Information from website only:

- Royal College of Anaesthetists (RCoA)
- Royal College of Obstetricians and Gynaecologists (RCOG)

Information derived from website and face-to-face discussion:

- Royal College of Psychiatrists (RCPsych)

Obituaries are not about death, although that is the hook for publication, giving a sense of newsworthiness. By contrast, biographies may be published during a person’s lifetime or long after their death, the latter form being found in, for example, the *Oxford Dictionary of National Biography.* ^12^ This paper includes both timely obituaries and short biographies written much later, as the Royal Colleges use both to record past lives. How to ensure that obituaries are created in a way likely to be helpful to bereaved people, and to best inform colleagues and the wider public today, as well as providing an honest record for historians, genealogists and other researchers in the future, is far from straightforward.

## Obituaries: creation, style and meaning

Since the mid-1980s, rather than just a factual narrative, obituaries in British newspapers have become an interpretation of the life and career of the deceased, often written by someone who knew him or her well.^13^ These obituaries are a way of acknowledging what the subject did in their life as well as how they did it – their values, principles, beliefs, spirit and style. Many of the so-called objective ‘facts’ found in obituaries are selected, constructed and curated for the purpose. They may tell us little about the person, may even be misleading and are open to interpretation alongside more subjective evidence. The tendency, for example, to credit hundreds of academic papers to clinicians and scientists reveals neither their actual contribution to the authorship, nor how they collaborated with their team, perhaps nurturing and inspiring, or possibly domineering and bullying.

Someone reading an obituary should be able to imagine being in the company of the person being described, despite never having met them, or even heard of them. Ann Wroe, obituaries editor of *The Economist*, commented that she writes obituaries:

‘for the story, and not whether the person is famous. I love it when someone’s had a quirky career […] I really try to get into the head of the person. I don’t want to write what everyone else thinks of the person. I want what they thought of the world. It’s a completely different viewpoint’.^14^

A good story may encourage readers to reflect both on the life of the deceased and on their own lives. *The Economist* has had only three obituaries editors since 1995: ‘It’s such a nice job that nobody wants to give it up,’ said Wroe.^14^ The positives of the role might also echo with *BJPsych Bulletin* past obituaries editors: Henry Rollin (1982^15^–2014) and Philip Graham (2014–2024).

If obituaries are a form of storytelling, we need to consider the art of writing them. In 1995, Stephen Lock, while *BMJ* obituaries editor, advised on the importance of ‘giving the truth about a rounded human being with his or her foibles as well as achievements. [This is] much more interesting and readable than the conventional honeyed platitudes’.^16^ He also advised avoiding ‘prolixity, cliche, and a curriculum vitae’ and euphemisms, stock phrases and superlatives (e.g. ‘best’; ‘most popular’; ‘beloved by all’; ‘had all the time in the world for’). They are unlikely to be true, and their repetition may have ‘a stultifying effect on readers’.^16^ They were more common in obituaries of the past, sometimes ‘kindly lies’ as Thomas Bewley (President, RCPsych 1984–1987) called them.^17^ Lock and Richard Smith, then *BMJ* editor-in-chief, commented: ‘The best obituaries portray people as they really were and as they would have wished to be remembered. That is what makes an obituary section compulsive reading’.^18^

Faced with increasing numbers of obituaries and a limited number of pages, in 1995 the *BMJ* circulated a questionnaire to over 1000 doctors asking their views. Seventy-five per cent replied. Most wanted obituaries kept, including giving space to ‘ordinary’ doctors. One-third wanted more attention paid to the subjects’ failings. Furthermore, ‘“top” or “famous” doctors should not necessarily get a longer obituary than “ordinary” doctors, whose lives may make much more interesting reading’.^18^ People pursue their careers influenced by numerous factors, and ordinary people can have extraordinary and inspiring stories. These are important matters at a time when equality, diversity and inclusivity are cardinal principles. Jane Calow commented in the journal *Mortality* that the terrorist attacks of 9/11 (2001) and 7/7 (2005) brought the memorialisation of ordinary people even further to the fore.^19^ *The Guardian* ‘Other lives’ obituaries series began in 2005, about the lives of extraordinary ordinary people who deserve noticing, ‘people less in the public eye, or lives lived beyond formal recognition’.^20^

As well as creating a more eclectic mixture of obituaries, another shift in the national press has been away from solemnity: obituaries ‘have become a source of daily fascination and delight […] [They are] anecdotal, discursive, yet elegantly concise; learned, touching and, in a kindly way, often extraordinarily funny’.^21^ The principle of *de mortuis nihil nisi bonum* (of the dead, nothing but good) has ceased to be so dominant and ‘The modern rule is that the frankness of the obituary should match, but not exceed, the frankness with which the subject conducted himself in life’.^21^ The need for honesty, plus an in-depth knowledge of the subject, links to the tradition of obituary authors’ anonymity.^8^ A signed obituary, especially by someone who knew the deceased and their family personally, may be too reverential, favouring ‘clichés of the funeral eulogy type and glossing too cosily over the more complex aspects of the life in question’.^21^ *The Times* has rigorously maintained the tradition of obituary author anonymity, but other newspapers have not. At various points in their history, medical journals have followed the anonymity etiquette, or appended an author’s initials, which often failed to conceal their identity, especially from colleagues. Those initialled or anonymous medical obituaries were often accompanied by a separate, signed tribute or appreciation.

## Medical Royal Colleges’ obituary processes and practices, past and present

The medical Royal Colleges approach obituaries in different ways. Decisions of policy and practice may be taken primarily by an individual or a committee. Some colleges’ websites provide valuable practical guidance, such as advising a potential author to inform the college of their intention to write the obituary, to avoid multiple submissions and possible rejections. Colleges differ in the level of collaboration and consent they require from a family to decide on publication, at one extreme publishing an obituary only when the family agrees, so if no one is contactable, the obituary is not published. Others advise contacting the family in the course of writing, but little more. Bereaved people are, of course, among the readership of obituaries, and although *de mortuis nihil nisi bonum* may be ‘out’, *primum non nocere* (first do no harm) is still ‘in’.

One college that never publishes obituaries, when asked why, answered: ‘We just never have’. Others, including the RCPsych, have had interludes (1971–1982) of not producing them.^17^ Some have uninterrupted traditions of honouring individuals, with their volumes now morphed into electronic formats. The RCP’s *Munk’s Roll* was initially compiled by Dr William Munk (1816–1898), who researched physicians back to the college’s foundation in 1518. It records the lives of Fellows, including those accused of malpractice or infringing RCP regulations. Now digitised and called *Inspiring Physicians*, it is a vast historical medical treasure trove.^22^ Similarly, the RCS has *Plarr’s Lives of the Fellows*.^23^ Starting from 1843, when the fellowship was introduced, it is now digitised and has over 10 000 biographical entries.^24^ Both aim to be fully inclusive of all those who hold their fellowships, although that term has different meanings: for the surgeons it is a post-graduate qualification to allow specialist practice, and for the physicians it is a mark of achievement awarded at a later career stage.

Today, the RCP and RCS publish obituaries in printed magazine or journal format only in exceptional circumstances. The RCP created a commemorative magazine about the lives of their physicians who died due to COVID-19,^25^ and the RCS published an obituary of Dame Clare Marx in their *Bulletin*.^26^ Other colleges, at the discretion of their honorary officers, might produce an informal tribute in a paper publication when a particularly eminent member dies. Unusually, the RANZCP does not limit the number of obituaries published in *Australasian Psychiatry*, although it has not yet introduced a website compilation. Online publication allows for corrections, revisions, updates or expansions of entries, processes encouraged by some colleges and appreciated by families and historians.^24,27^ Bringing obituaries together online may make them more widely accessible, rather than obscured by journal paywall or passworded members-only access.

Royal College obituaries come about in various ways, which may also influence their style. There is a Royal College tradition, including at the RCPsych, that the president sends a condolence card or letter to the deceased’s relatives. Those sent by the RCP president also mention *Inspiring Physicians*, by way of invitation to contribute, whereas those from the RCPE formalise their invitation on a small card inserted into the president’s condolence card:

‘The College, in some instances, creates tributes and obituaries to be published on our website. If you would like to provide us with information to support this or discuss this further, please contact [name: email/phone].’

Ideals may have to be balanced against resources regarding the extent of coverage and website or print publication, and the RCPE’s wording probably reflects this. Nevertheless, our survey respondent noted, so far, they ‘have been able to support all who have got in touch’. Recognising the need for resourcing is important. To ensure that obituary or biographical databases are kept up to date is time-consuming. The RCP and RCS, for example, each typically check, edit and upload over 100 entries annually. Carrying out and coordinating the work may fall to paid staff, including archivists, librarians, and public engagement and communications teams. There is also some inter-collegiate cooperation: the RCP has reciprocal publishing agreements with other Royal Colleges for commemorating those who were fellows of more than one college.

Generally, Royal College obituaries are written by friends, relatives or colleagues who knew the deceased personally. Some colleges also commission the services of a freelance professional obituaries writer or have someone ‘in-house’ to take on the task if that is not possible. Some colleges also collect biographical information to facilitate obituary creation. One stores ‘unpublished electronic records (with family’s permission) for future researchers and eventual submission to our online resource of historical members’. The RCoA also seeks out information, encouraging retired fellows to complete a ‘Lives’ form^27^ or submit a *curriculum vitae* to the archivist and assuring that ‘the document will not be released to anyone prior to your death without your clearly stated permission’. The RCoA is then able to create an entry for its *Lives of the Fellows* if no one else is able to do so. However, a form may be associated with a formulaic, rigidly structured obituary, tending to focus on what a person did in their career, ‘facts’ more than the hows and whys of happenstance, and what made that individual tick. They risk being depleted of anecdote and humour, and lacking in the eccentricities, struggles, successes and quirks that convert a *curriculum vitae* into a gripping, true, life story.

## Conclusions

As the *BJPsych Bulletin*’s obituaries editor, I want to hear and tell meaningful life stories of our members and preserve those stories as part of the profession’s heritage. As psychiatrists who take account of our patients’ life histories and may have supported people with complex or prolonged grieving, we have insights that may help us create obituaries for many, if not all, former College members, whether in print or on a website. Arguably, extending on from our professional skills and interests, the RCPsych should have the most meaningful narratives and comprehensive coverage of all the medical Royal Colleges. I am not convinced that we do just yet, but perhaps this short paper will be a starting point for further discussion.

If we aim to achieve ‘compulsive reading’ status for RCPsych obituaries and value the principles of equality, diversity and inclusion, we need to consider both the literary style and the stories behind the multiplicity of ways in which psychiatrists pursue their careers. It is important to know about those people who reached the pinnacle of the profession, whose successes most of us will never be able to emulate. However, it is equally important to record the lives of those who did not achieve accolades usually bestowed in later life, such as those who sadly die young; and those who overcame hurdles of disadvantage, took unconventional career routes, or somehow went above and beyond the call of duty; or clinicians, dedicated to their patients, who kept far from research participation, national psychiatric politics and the public gaze. They may be the ones with the most inspiring and compelling life stories. Perhaps, while hoping to live a long, productive and fulfilled life, we should be asking ourselves how we would like our life stories to be written when our time comes.

## Data Availability

Data, anonymised where appropriate, that support the findings of this study are available from the author, on reasonable request.

## About the author

**Claire Hilton**, MA, MD, PhD, is the obituaries editor of the *BJPsych Bulletin* and honorary archivist at the Royal College of Psychiatrists, London, UK.
